# The Use of Fish Oil Lipid Emulsion in the Treatment of Intestinal Failure Associated Liver Disease (IFALD)

**DOI:** 10.3390/nu4121828

**Published:** 2012-11-27

**Authors:** Melissa I. Chang, Mark Puder, Kathleen M. Gura

**Affiliations:** Boston Children’s Hospital, 300 Longwood Avenue, Boston, MA 02215, USA; Email: melissa.chang@childrens.harvard.edu (M.I.C.); mark.puder@childrens.harvard.edu (M.P.)

**Keywords:** parenteral nutrition, pediatrics, lipid emulsions, intestinal failure-related liver disease, fish oil

## Abstract

Since 2004, fish oil based lipid emulsions have been used in the treatment of intestinal failure associated liver disease, with a noticeable impact on decreasing the incidence of morbidity and mortality of this often fatal condition. With this new therapy, however, different approaches have emerged as well as concerns about potential risks with using fish oil as a monotherapy. This review will discuss the experience to date with this lipid emulsion along with the rational for its use, controversies and concerns.

## 1. Introduction

Parenteral nutrition (PN) provides a life-saving intervention for patients unable to absorb enteral nutrients secondary to intestinal failure due to inadequate bowel length or function [1]. However, over the course of its use, the development of intestinal failure-associated liver disease (IFALD) has been the source of significant morbidity and mortality. Up to 60% of infants undergoing prolonged courses of PN are afflicted with this disease. IFALD can progress to varying degrees of hepatic steatosis, hepatocellular injury, cholestasis, with ultimate liver failure and death [2]. The severity of liver disease and presence of cholestasis correlates with survival [3,4]. Overall, 15% of these patients with cholestasis will progress towards end-stage liver disease [3,5,6,7]. Without transplant, mortality in this population approaches 100% [5]. The defining mechanism in which intravenous fat emulsions (IVFE) are responsible for the development of IFALD remains unresolved. However, many risk factors have been identified including: prematurity, low birth weight, prolonged course of PN, intestinal stasis complicated by bacterial overgrowth, catheter related sepsis, and the diagnosis of gastroschisis or jejunal atresia [8]. Of these factors, the most important has been considered prematurity secondary to the underdevelopment of the hepatobiliary system. Catheter-related sepsis has been determined to be the second most important factor as repeated episodes of sepsis have shown to have a 30% increase in serum bilirubin [8,9].

Recent studies have demonstrated that the use of parenteral fish oil lipid emulsions (FO) rather than the commonly used soybean oil lipid emulsion (SO) has conferred significant recovery of biochemical cholestasis and thereby reduction in morbidity and mortality in patients with IFALD. This review provides an overview of the use of FO in the clinical setting, and the potential mechanisms for its efficacy in treating IFALD.

### 1.1. Essential Fatty Acids (EFAs)

**Figure 1:**
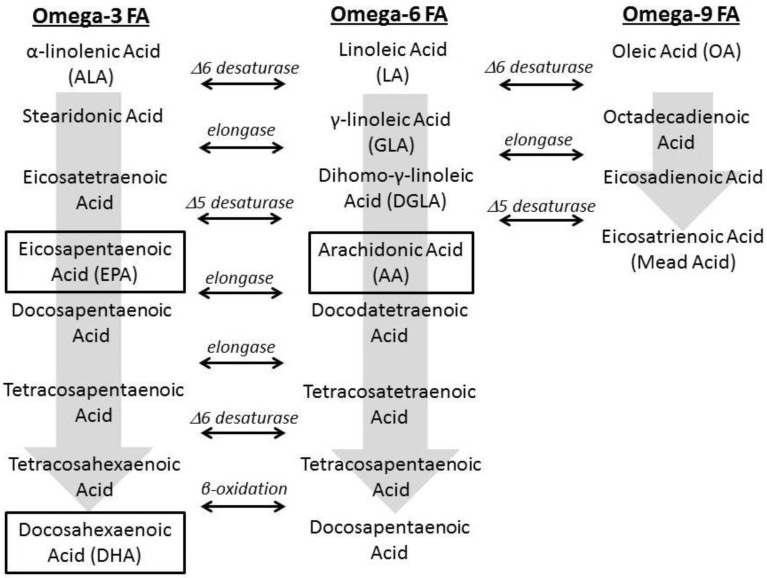
Docosahexaenoic acid (DHA) and arachidonic acid (AA) Pathways (adapted from Friedman 2006 [14]).

In 1929, Burr and Burr first observed that dietary fats are essential for normal growth and development in animals [10]. Fatty acids play a diverse role in human body, including: maintaining cellular membrane integrity; contributing as a major component in phospholipids, triglycerides, and cholesterol esters; and acting as a biochemical mediator in cell signaling. There are 3 major types of fatty acids in mammalian cells: omega-3, omega-6, and omega-9. Omega-3 and omega-6 fatty acids are categorized as essential and require dietary intake, as mammals cannot insert the double bond at position-3 and -6 to synthesize α-linolenic acid (ALA) and linoleic acid (LA), respectively. Holman *et al.* first described essential fatty acid deficiency (EFAD) manifesting as impaired growth, dermatitis, hepatic steatosis, renal toxicity, and pulmonary abnormalities [11]. This condition which occurs from low dietary intake, severe malabsorption, or increased physical requirements seen in growth, became clinically relevant with the availability of fat-free parenteral nutrition [12]. Tashiro initially described the prevention of EFAD in four PN-dependent pediatric patients when they are administered at least 2% of their total calories as LA [13]. In normal fatty acid metabolism, elongases and desaturases convert LA to arachidonic acid (AA, tetraene) (Figure 1) [14]. In EFAD, the body stores of omega-3 and omega-6 fatty acids are depleted, thereby resulting in a decrease of AA and subsequently an increase in *de novo* lipogenesis (DNL). Oleic acid (omega-9), which can be endogenously produced, via elongation and desaturation, form Mead acid (triene). This mechanism is necessary to maintain the number of double bonds in the cell membrane to provide structural integrity [15]. Therefore, the biochemical definition of EFAD is the serum triene:tetraene ratio >0.2, where the absence of ALA and LA has resulted in compensatory production of Mead acid from oleic acid [11]. Therefore, human studies have shown that the optimal daily requirements of LA are 1%–3% of the total caloric intake in order to prevent EFAD [16,17]. 

### 1.2. Fatty Acid Regulation

Dietary fat is an essential part of the human diet that is metabolized and stored by the liver. In conditions such chronic consumption of excessive calories (overfeeding) or impaired fatty acid metabolism, the accumulation of lipid results in hepatic steatosis [18]. Increased lipid content resulting in deregulation of hepatic lipid metabolism can arise from: excessive peripheral triglycerides (TG) stored in adipose, dietary TG (chylomicrons) in overfeeding, decreased export of lipids from the liver as very low density lipoprotein (VLDL), increased *de novo* lipogenesis, and reduced β-oxidation of fatty acids [19]. While excessive saturated fat (SFA) promotes lipid storage and inflammation, polyunsaturated fatty acids (PUFAs), in particular omega-3 FA, play a hepatoprotective role. Omega-3 FA reduce synthesis and oxidation of saturated fatty acids (SFAs) and monounsaturated fatty acids (MUFAs), lowering hepatic fat content, and thereby preventing accumulation and eventual steatosis [20]. 

At the synthetic level, DNL and MUFA synthesis are intricately controlled by fasting and feeding states, insulin, carbohydrates, and dietary fat [21]. The DNL and MUFA synthetic enzymes are regulated by key transcription factors including sterol regulatory binding protein-1c (SREBP-1), carbohydrate response element-binding protein (ChREBP), and peroxisome proliferator receptor α (PPAR-α). Elevated SREBP-1 induces DNL, MUFA, and TG synthesis and storage. Diets supplemented with PUFAs, specifically docosahexaneoic acid (DHA, omega-3 FA), suppress the amount of SREBP-1 by reducing gene transcription, mRNA turnover, and accelerated degradation of its mature form. Therefore the enzymes involved with DNL and MUFA synthesis are similarly affected and downregulated [20]. High-carbohydrate/low-fat diets promote the accumulation of nuclear ChREBP thereby inducing DNL and MUFA synthesis. PUFA supplementation suppresses the presence of ChREBP, resulting in the downregulation of its target genes [20]. PPAR-α is a fatty acid activated nuclear receptor responsive to changes in intracellular fatty acids levels [22]. PPAR-α is a key regulator of mitochondrial, peroxisomal, and microsomal fatty acid oxidation genes, as well as those involved with gluconeogenesis [23,24]. Eicosapentaenoic acid (EPA, omega-3 FA) is a potent activator of PPAR-α, whereas SFA, MUFA, and other PUFAs only exert a mild response. Hepatic steatosis is associated with low levels of β-oxidation; the presence of EPA upregulates β-oxidation via PPAR-α, thereby preventing hepatic lipid accumulation [20]. 

At the consumption level, omega-3-rich FO, decreases fasting and postprandial TG levels independent of the fat composition of the test meal given; this effect is hypothesized to be due to accelerated chylomicron clearance via lipoprotein lipase [25]. VLDL production in the liver is dependent on the presence of triacylglcerol and apolipoproteins required for assembly. VLDL secretion is inhibited by omega 3-FA via its downregulation of DNL, upregulation of fatty acid oxidation, and promotion of apolipoprotein degradation [26,27,28,29]. 

### 1.3. Role of Fatty Acids in Inflammation

**Figure 2:**
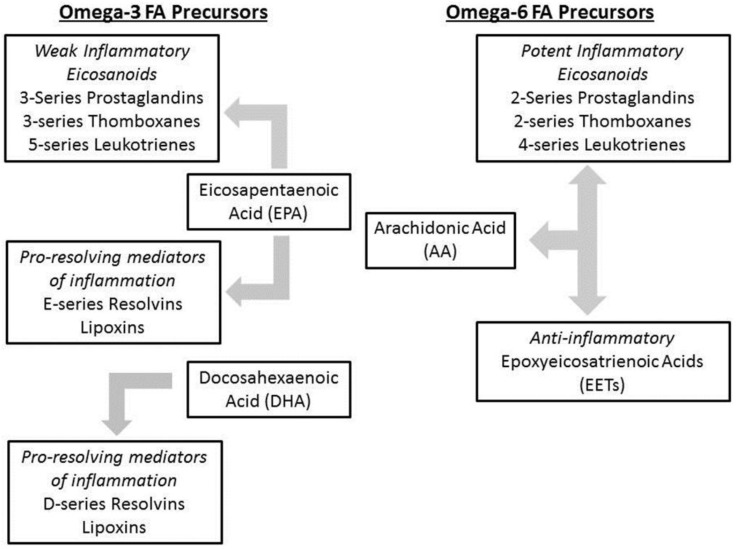
Role of FA in Inflammation.

The aforementioned omega 3 and omega 6 FAs are important precursors of eicosanoids and prostaglandins, which are critical in various biochemical pathways (Figure 2), including inflammation [30]. Excess intake of omega-6 FA, especially LA, leads to higher levels of inflammatory mediators (e.g., nuclear factor-κB, interleukin 6, tumor necrosis factor-α) [31,32]. AA, which is the major downstream product of LA, is a key substrate for 2-series prostaglandins and thromboxanes via the cyclooxygenase (COX) pathway, and 4-series leukotrienes via the lipoxgenase (LOX) pathway [33]. These lipid mediators promote inflammation by secreting interleukin-6 (IL-6), involved in leukocyte chemotaxis and vasodilation [34]. The most potent of these mediators, prostaglandin E2 (PGE2) and leukotriene B4 (LBT4), are involved in chronic inflammatory processes such as asthma, rheumatoid arthritis, and ulcerative colitis [35]. While AA plays an important role in inflammation, it also acts as a precursor for epoxyeicosatrienoic acids (EETs) via cytochrome 450 pathway. EETs promote anti-inflammation by decreasing leukocyte adhesion to the endothelial cell surface and downregulating NF-κB [36,37]. The AA-derived precursor gamma linolenic acid (GLA) further complicates this balance, as when GLA is taken as a supplement, it is largely converted to dihomo-γ-linolenic acid (DGLA), which is also a competitor of AA. Moreover, GLA is a precursor of the less pro-inflammatory 1-series prostaglandins which are similar to 3-series prostaglandins [38].

ALA forms the downstream product EPA, including 5-series leukotrienes and 3-series prostaglandins (prostaglandin E_3_, thromboxane A_3_), and their synthesis is mediated by the same enzymes. EPA, the downstream product derived from ALA, is critical in the formation of 3-series prostaglandins and thromboxanes via the COX pathway, and 5-series leukotrienes via LOX [39]. EPA acts as a competing substrate for COX and LOX. ALA and other omega-3 FA also inhibit the activities of omega-6 and omega-5 desaturases on LA, reducing the production of AA [40]. These mediators tend to be more anti-inflammatory in contrast to those formed from AA, thereby shifting towards a more anti-inflammatory state in the presence of these EPA derived mediators. Moreover, EPA and its derivative, DHA, bind to PPARs α/γ and G-protein coupled receptors (GPR) 120 and 40. This relationship results in the overall downregulation of NF-κB, thereby attenuating the inflammatory cascade [34,41]. This occurs by DHA and EPA binding to PPAR-γ and GPR-120 resulting inhibited degradation of IκB (the inhibitory subunit of NF-κB) by decreasing phosphorylation of the product, reducing NF-κB. In addition, the bound omega-3 FA-PPAR-γ unit can directly interact and inhibit NF-κB by preventing nuclear migration and transcription [42,43].

In simple terms, AA (omega-6 derived) products are considered to be pro-inflammatory mediators whereas EPA (omega-3 derived) products are anti-inflammatory, or rather less pro-inflammatory [44,45,46] which makes their interrelationship critical, especially given that AA and EPA are competitive substrates [47]. 

The resolution of inflammation has been recently studied as a dynamic process. These processes are mediated by biochemical factors known as specialized pro-resolving mediators (SPMs) that are derived from PUFAs including: lipoxins derived from AA; resolvins derived from EPA and DHA; and protectins and maresins that are derived from DHA [48,49]. Upon neutrophil interaction with endothelial cell surface, EPA and DHA are converted to E- and D-series resolvins via cytochrome P450, 5-LOX, 15-LOX, and COX-2, enzymes upregulated during inflammatory states. These SPMs produced inhibit the production of inflammatory leukotrienes, prostaglandins, and interleukins (including the previously described IL-6, PGE2, LBT4), and prevent further neutrophil recruitment. These mediators play an important role in promoting resolution of various inflammatory mediated disease processes (e.g., acute lung injury, peritonitis, acute kidney injury, postoperative pain, and retinopathy) [50,51,52,53,54,55]. The protective effect of these factors was demonstrated in the intraperitoneal administration of resolving E1 in ob/ob mice, the commonly used murine model for insulin resistance and hepatosteatosis. The treated mice were found to have overall lower transaminase levels (as a marker of liver injury), and decreased F4/80 immunostaining (macrophage marker) [56]. FO lipid emulsions are high in the omega-3 fatty acids EPA and DHA in contrast to the traditional SO, therefore suggesting that this supplementation also plays a role in an anti-inflammation and hepatoprotection.

### 1.4. Intestinal Failure Associated Liver Disease (IFALD)

For over 50 years, the nutritional needs of a majority of PN patients have been met with standard SO-based IVFE. Morbidity was recognized after a decade of clinical use when the use IVFE moved away from EFA supplementation and was instead used as alternative source of non-protein calories, as excessive intake of parenteral dextrose was determined to be detrimental. Over time, the provision of higher doses of SO IVFE (*i.e.*, 30 to 60 g per day in adults or 3–4 g/kg/day in infants) compared to the doses recommended for its original indications to prevent EFAD (*i.e.*, 50 g per week in adults or 0.25 g/kg/day in children) may have potentially predisposed patients to additional complications, including hepatic dysfunction and exaggerated systemic inflammatory response in the critically ill [57,58,59,60,61,62,63].

Multiple hypotheses exist to explain the pathogenesis of IFALD including: altered gut hormonal profiles [8], the propensity for bacterial translocation in the absence of enteral intake [64,65], intestinal stasis resulting in the reduced clearance of hepatic bile acids [64], and direct deficiencies or toxic components of the PN solution itself [66,67]. The suspected toxic components have included the high dextrose load, certain amino acids in the PN solution, and the use of lipid emulsions. 

Early stage liver injury is speculated to be hepatic steatosis with progression onto steatohepatitis. This is mediated via tumor necrosis factor α (TNF-α) and other inflammatory mediators released by Kupffer cells during sepsis and/or inflammation related to native disease or surgery [68]. While several hypotheses have been proposed to explain the pathogenesis, none have been conclusive to-date. One theory is the “two hit” hypothesis, where the first hit involves the development of hepatic steatosis [69] rendering the liver more susceptible to a second, as yet undefined, hit, resulting in more severe liver damage [68,70,71]. The first hit can result from an imbalance in the rate of entry, synthesis, and clearance of fat from the liver, which may be contributed by parenteral carbohydrate load [72], and the administration of IVFE [73]. More specifically, the uptake of TGs and fatty acids, the rates of re-synthesis and DNL of fatty acids and TGs, the secretion of these compounds via plasma or bile, or oxidation of fatty acids may be altered [71,74]. 

During the early stages of liver injury, hepatocytes are exposed to TNF-α and Fas ligands (Fas-L). These molecules activate caspase pathways that lead to cell death [75]. TNF-α itself is instrumental in the production of insulin resistance, which is common in IFALD [76,77]. Hepatocyte exposure to TNF-α initiates intracellular signals that may lead to the release of reactive oxygen species and increased mitochondrial permeability [78,79]. Oxidation of the fat via free radicals, in response to inflammation, is thought to contribute to the later changes of steatohepatitis and cholestasis. Healthy hepatocytes respond to this insult by activating transcription factors such as NF-κB and adaptor protein 1 that can lead to adaptation and survival by synthesizing anti-apoptotic proteins such as Bfl-1 and oxidant-detoxifying enzymes such as manganese superoxide dismutase [80]. Less viable, fatty hepatocytes, however, are not as resilient and may react to TNF-α by activating the caspase pathway leading to apoptosis [75,81] or by inducing sphingomyelinase and thereby increasing lipid peroxidation resulting in hepatocyte necrosis. 

IFALD is characterized by fatty deposits and diffuse hepatocyte ballooning [82]. This may progress towards bile duct proliferation and cirrhosis [64]. Major serum enzyme markers for liver injury include alkaline phosphatase (AP), aspartate aminotransferase (AST), alanine aminotransferase (ALT), and direct bilirubin levels. Elevations in direct bilirubin are a relatively specific marker for initial cholestasis, commonly defined as greater or equal to 2.0 mg/dL. Persistent elevation may subsequently reflect ESLD. Kaufman *et al.* determined that worsening hyperbilirubinemia, thrombocytopenia, and hypoalbuminemia were all independent risk factors of liver failure [83]. Once significant fibrosis occurs, portal blood flow is compromised and portal hypertension ensues. This disruption in portal flow produces the manifestations of ESLD including gastrointestinal bleeding, jaundice, ascites, hepatosplenomegaly, malnutrition, and severe pruritus. This hepatic insult may ultimately require a combined liver and small bowel transplant. Liver and small bowel transplant is associated with a substantial morbidity and mortality, requiring lifelong immunosuppression [84]. 

### 1.5. Lipid Emulsions and IFALD

Previously, PN-dependent patients were supplemented with SO to prevent EFAD, however, increasing evidence suggests the potential toxicity of this oil source and its role in IFALD [85]. In the last decade, the use of FO for lipid supplementation has investigated due to its high omega-3 content, reduced omega-6 fatty acid content, and absence of phytosterols. Practitioners outside the United States have had clinical access to a multitude of IVFEs that combine SO with a variety of oils including olive, fish or MCT oils, resulting in products that are metabolized via different pathways. By formulating an IVFE using these alternative fat sources, one could produce a product that was of equal caloric value that potentially reduced in the pro-inflammatory properties of SO. Subsequent modifications in lipid emulsions have continued to focus on reducing the overall omega-6 FA content in hopes of creating an IVFE that is better tolerated by critically ill patients. Unlike other oils, FO is rich in omega-3 FA, which as discussed previously, is more bioactive in comparison to olive or MCT oils, offering the advantage of possessing more favorable nutritional and pharmacological properties. 

EFAD can result in hepatic steatosis [86], and by reversing biochemical EFAD, hepatic steatosis can be prevented [87]. More recent evidence has demonstrated that lipids are metabolized differently depending on their route of administration. Enteral lipids are absorbed by the enterocyte in the form of a micelle and packaged into chylomicrons for ultimate disposal in the liver. Once in the bloodstream, these particles rapidly acquire apolipoproteins from circulating high-density lipoproteins and can subsequently be metabolized by the liver. The emulsified particles of commercially made and intravenously administered SO IVFE, mimic the size and structure of chylomicrons, but differ in their content. In contrast to chylomicrons, artificial lipid particles primarily contain EFAs and omega-6 TG, devoid of cholesterol or protein. Additional evidence has suggested that these omega-6 fatty acid-containing IVFE are dependent on lipoprotein lipase, apolipoprotein E, and low-density lipoprotein receptors for clearance, and are metabolized with less lipolysis and release of EFAs than chylomicrons. In fact, it appears that they may be cleared as whole particles by tissues other than the liver [66]. In Javid *et al.*, a murine model of hepatic steatosis was created by inducing EFAD in mice by providing them with a high carbohydrate diet (HCD) for 19 days [88]. During this time, mice received SO either enterally or intravenously. Two dosing regiments of enteral lipid were used to investigate the possibility of a dose-dependent pattern. This study verified that enteral lipid supplementation was protective against hepatic steatosis in a dose-dependent manner, whereas persistent and severe steatosis was seen with IV lipid administration. No difference in the amount of lipid administered or the EFA status between the high dose enteral and the IV groups, therefore, the liver injury was secondary to the route of administration. On magnetic resonance spectroscopy, administration of PN without lipid supplementation in comparison to control mice revealed a significant increase in liver fat content (15.78% ± 3.74% *vs.* 5.39% ± 1.10%, *P* < 0.001). Comparing the low *vs.* high dose enteral group, there was also a significant decrease in fat content (19.34% ± 4.32% *vs.* 8.08% ± 4.28%, *P* = 0.093). Most importantly the intravenous lipid group was also significantly greater than the high dose enteral group (18.13% ± 4.84%*vs.* 8.08% ± 4.28%, P < 0.001). These factors may account for the increased incidence of steatohepatitis associated with intravenous infused SO IVFE [88].

In addition to oils and emulsifying agents, there are other components that may be considered significant when considering different lipid emulsions. For example, phytosterols found in SO are thought to have a deleterious effect on hepatic function [89]. Phytosterols are plant-derived lipids structurally similar to cholesterol; however, enteral absorption is limited to 5%–10%, approximately 1/10 that of cholesterol [90,91,92]. In humans, >95% of dietary phytosterols are excreted in feces via the enterocyte apical ABCG5/G8 transporter, preventing entry into systemic circulation. In parenteral administration, phytosterols cannot be converted to bile acids, thereby limiting their excretion. Instead, these plant sterols are secreted into the liver via a hepatocyte canalicular membrane ABCG5/G8 transporter, where they are slowly metabolized [93,94]. 

Phytosterols are particularly pronounced in SO (Table 1). Phytosterol administration in neonatal piglets was shown to increase serum bile acids and reduce bile-acid dependent bile flow; and only phytosterols, and no other component of PN, was shown to predispose neonatal piglets to developing cholestasis. Newborn piglets receiving phytosterol-free FO maintained normal bile flow and liver function tests, whereas those given SO that is rich in phystosterols developed cholestasis and impaired bile flow rates [95,96,97]. Patients with IFALD showed a direct relationship between serum phytosterol levels and the severity of IFALD. In clinical studies, patients with normal livers had a phytosterol level of <0.06–0.1 mmol/L, whereas those with mild cholestasis and severe cholestasis had values of <0.3 and 1.3–1.8 mmol/L, respectively [91]. The long term use of SO may also lead to progressive accumulation of phytosterol content in cell membranes and plasma lipoproteins, which has been associated with cholestasis in PN dependent children [97]. 

**Table 1:** Comparison and characteristics of four different lipid emulsions (10 g fat/100 mL solution) *.

Product	Intralipid	ClinOleic	SMOF lipid	Omegaven
Manufacturer	Baxter Healthcare/Fresenius Kabi	Baxter Healthcare/Parenteral SA	Fresenius Kabi	Fresenius Kabi
Oil source (g)				
Soy bean	10	2	3	0
Safflower	0	0	0	0
MCT	0	0	3	0
Olive oil	0	8	2.5	0
Fish oil	0	0	1.5	10
Alpha Tocopherol	38	32	200	150–296
Phytosterols (mg/L)	348 ± 33	327 ± 8	47.6	0
Fat composition (g)				
Linoleic	5	0.9	1.87	0.1–0.7
Alpha-linolenic	0.9	0.1	0.24	<0.2
EPA	0	0	0.24	1.28–2.82
DHA	0	0	0.22	1.44–3.09
Oleic	2.6	0.8	2.78	0.6–1.3
Palmitic	1	0.7	0.92	0.25–1
Stearic	0.35	0.2	0.27	0.05–0.2
Arachidonic	0	0.03	0.05	0.1–0.4

* All data has been provided by the manufacturer (Adapted from Meisel 2009 [62]).

Phytosterols include campesterol, sitosterol, and stigmasterol [98]. Stigmasterol has the highest concentration in SO, and is an antagonist of Farsenoid X Receptor (FXR) [99]. FXR normally suppresses 7 alpha-hydroxylase, the rate limiting enzyme in bile acid synthesis [100,101]. In studies using a murine model for cholestasis, mice given GW4064, a synthetic FXR agonist, were found to have reductions in ALT, AST, lactate dehydrogenase, alkaline phosphatase, bile acids, and bilirubin levels [102]. Therefore, these findings suggest that phytosterols contribute to cholestasis by downregulating suppression of bile acid synthesis [99].

Similarly, the amount of α-tocopherol (vitamin E) present in an emulsion has been considered another critical factor and difference between SO and FO lipid emulsions [103]. Oxidative stress occurs when cellular byproducts of reactive oxygen species and hydrogen peroxide are not utilized or neutralized within the cell [104]. Alongside antioxidant enzymes (e.g., superoxide dismutase, catalase, glutathione peroxidase), antioxidants such as glutathione, tocopherol, and ascorbic acid, scavenge for these pro-oxidant species and neutralize them into stable products [105]. Oxidative stress has been proposed as the “second hit” in hepatosteatosis leading to cellular injury and hepatic apoptosis secondary to abnormal fat accumulation [69]. Rabbits receiving PN with SO, when compared to the control group fed chow with IV saline, were found to have an increase in bilirubin, steatosis, and inflammatory cell infiltrate after 10 days of treatment. Furthermore, these findings were found to have decreased dismutase activity, increased lipid peroxidation and apoptosis, all indicative of increased oxidative stress [106]. In comparison to FO, SO has less α-tocopherol (Table 1). Furthermore, prolonged use of SO is thought to deplete the antioxidant defense secondary to depletion of tocopherol content in plasma lipoproteins. While tocopherol has not been specifically studied in relationship to IFALD, mice supplemented with tocopherol displayed improvement of hepatosteatosis in murine models treated with lipopolysaccaharide [107]. Thus, tocopherol, which is found in large quantities in FO, may act as a potent antioxidant in hepatosteatosis, protecting the liver from further oxidative injury [108,109]. 

Whereas the benefits conferred from tocopherol administration has not been studied in IFALD, phase 3 multicenter randomized controlled studies have been conducted in adults and children evaluating the efficacy of tocopherol in non-alchoholic fatty liver disease (NAFLD) [110,111]. Although the etiology of this disease is thought to be secondary to insulin resistance and overall metabolic syndrome, NAFLD is a clinicopathological diagnosis demonstrating hepatic steatosis with potential progression to cirrhosis. Despite there being no significant difference in serum biochemical markers and histological findings in children, there was a statistically significant improvement in histologic hepatocellular ballooning and resolution of non-alcoholic steatohepatitis (NASH). Adult subjects exhibited similar improvements in histologic features as well as statistically significant reductions in histologic lobular inflammation, steatosis, as well as serum AST and ALT. Study differences in outcome are speculated be attributed to the difference in distribution and severity of fat and inflammation, and/or the overall etiopathogenesis of disease. The benefit in NAFLD-affected children remains unclear. Likewise, treatment with tocopherol administration in the IFALD population remains unknown, however, given the overall improvement in histologic hepatic ballooning and resolution of NASH in NAFLD-affected children, further investigation of tocopherol in this population may be warranted.

In order to gain further insight as to the impact of IVLE formulation on hepatic steatosis, Meisel *et al.* compared five commonly used IVLEs in the clinical setting (Table 1) [62]. In this study, mice were given a fat-free, HCD enteral diet similar to PN solution which supplemented with one of the currently available types of IVLEs (*i.e.*, 100% SO, 50% SO/50% safflower oil, 80% olive/20% SO, 30% SO, 25% olive, 15% FO and 30% MCT, or 100% FO). Of these five separate IVLEs, only the livers of the mice in the FO monotherapy group appeared grossly normal with only a very mild amount of microvesicular fatty infiltration in comparison to the moderate to severe macro- and microvesicular steatosis seen in the other groups in histology. Magnetic resonance spectroscopy was utilized to quantitatively determine the fractionation of fat in these liver specimens. Mice on the standard control chow diet had a mean 3.5% ± 0.1%, whereas mice receiving HCD with intravenous saline had a mean hepatic fraction of 30.0% ± 2.5%. The mice receiving the SO, SO/safflower, olive/SO, SO/MCT/olive/FO IVLEs had hepatic fat fractions of 17.4% ± 3.6%, 21.9% ± 1.3%, 22.5% ± 1.4%, 12.6% ± 1.4%, respectively. The FO group had a mean hepatic fraction of 8.4% ± 1.1% [62]. 

**Figure 3:**
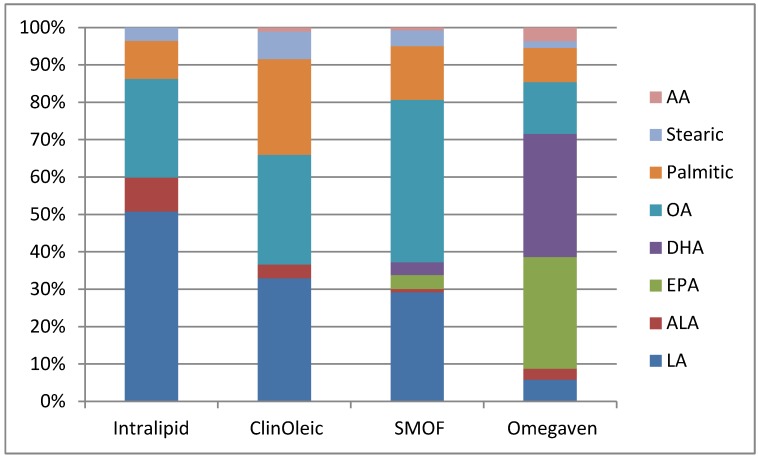
Fat composition based on percentage (Adapted from Meisel 2009 [62]).

Unlike SO which contains considerable amounts of its 18 carbon precursors, LA, and ALA, FO contains their downstream metabolites AA, DHA, and EPA (Figure 3). While concerns have emerged that FO given as an exclusive lipid source would result in possible EFAD, growth retardation, and delayed psychomotor neurodevelopment secondary to not containing adequate LA, multiple clinical and animal studies have confirmed no evidence of growth failure or EFAD [112,113,114,115]. Most recently, Nehra *et al.* demonstrated that a diet consisting of only DHA and AA was safe for long term consumption over 6 murine generations without any evidence of EFAD, dermatitis, or impaired growth [116]. Le *et al.* demonstrated that downstream metabolites of ALA, DHA, and LA, AA, can function as the EFAs [117]. Using a murine model of EFAD and consequent hepatic steatosis, the authors gave mice varying amounts of DHA and AA to determine whether exclusive supplementation of these specific fatty acids could prevent EFAD and inhibit or attenuate hepatic steatosis. They reported that mice supplemented with DHA and AA at 2.1% or 4.2% of their calories for 19 days had normal liver histology and no biochemical evidence of EFAD, which persisted when observed after 9 weeks. They concluded that supplementation with sufficient amounts of DHA and AA alone without the precursor ALA and LA acids met EFA requirements and prevented hepatic steatosis [117].

### 1.6. Clinical Experience

In July 2002, FO IVLE was first used as an “off label” monotherapy for the provision of EFAs. This was used in a PN-dependent child allergic to SO. He was therefore unable to tolerate the currently commercially available products. After months of receiving fat-free parenteral nutrition with intermittent enteral feeds, he developed EFAD manifested as a rash and an elevated plasma triene:tetraene ratio of 0.231. His signs of EFAD resolved on FO monotherapy with disappearance of the rash and normalization of the ratio [118]. As a result of this single case report, coupled with subsequent laboratory findings in murine models that SO IVLE may predispose patients to IFALD, the practice by which IVLE are used in children in the United States has undergone a major reevaluation.

Since 2004, FO IVLE monotherapy has been used infants with IFALD as a compassionate treatment of their liver disease. The original protocol had all forms of SO discontinued, and treatment with FO was initiated with a minimum dose of 1 g/kg/day, 5 times higher than the manufacturer’s labeled supplement dose of 0.2 g/kg/day [119,120,121]. In one case report, an infant with IFALD was treated with FO monotherapy dosed at 1.5 g/kg/day and it was well-tolerated [122]. In most instances, clinical improvement occurred approximately one month after initiation of treatment with subsequent resolution of biochemical cholestasis within 2–3 months [121,122,123,124,125,126]. While IFALD in adults is rare, several case reports have demonstrated similar reversal of cholestasis and histologic improvement upon transition from SO to FO IVLE [126,127,128]. To date, there has not been any randomized controlled trials conducted that compare equal doses of SO IVLE with FO IVLE in the treatment of pre-existing IFALD. In order for such a study to be performed, there would need to be randomization of subjects who develop IFALD while receiving SO IFVE to continue to receive the very SO IVLE that precipitated the condition. At the recent FDA GREAT Meeting discussing Parenteral Nutrition Associated Liver Disease in September 2012, such a study would be difficult to perform because it would it would be difficult to achieve equipoise given the increase use of FO monotherapy throughout the U.S.

Potential concerns with FO monotherapy have included EFAD as discussed previously. EFAD has not been observed although EFAD can occur if doses <1 g/kg/day are used. In one instance, EFAD developed in a study subject who had been weaned off PN but was still in the post-drug exposure monitoring period. The patient had a severe fat malabsorption condition that ultimately required the temporary use of pancreatic enzymes and the reinstitution of FO IVLE in order to correct the EFAD [120]. In another case series, EFAD was reported in subjects unable to receive a minimum of 1 g/kg/day of FO IVLE due to fluid restrictions and lack of adequate vascular access [121]. A case series by Gura *et al*. reviewed the clinical outcome of the use of FO IVLE in the treatment of IFALD [120]. Eighteen patients were compared to a group of historical controls described in Andorsky *et al*. [7]. The findings revealed that patients receiving the FO based monotherapy developed cholestasis reversal 4.8 times faster than the historical cohort receiving the traditional SO based lipid emulsion. Upon adjustment of baseline bilirubin levels, gestational age, and the diagnosis of necrotizing enterocolitis, the time to reversal was 6.8 times faster. There were only 2 deaths recorded in the FO monotherapy group *versus* 7 deaths in the soy-based group, and none of the FO treated cohort progressed towards liver transplant while 2 transplants occurring in the historical cohort. More importantly, despite prior concerns, FO monotherapy was not associated with the development EFAD, hypertriglyceridemia, coagulopathy, infections, or growth failure. In a separate study, data from 10 children who received no enteral intake, while completely dependent on PN and FO monotherapy for at least 1 month were reviewed for evidence of EFAD. Gestational age-adjusted Z scores for length, growth, and head circumference at baseline were also compared with the corresponding z scores. None of the patients developed biochemical or clinical evidence of EFAD. Moreover, the Z scores were not statistically different from those of the reference population, indicating no growth impairment [113]. In a follow-up study, the lipid and FA profiles of 79 PN-dependent children with IFLAD and dyslipidemia who had been treated with FO monotherapy were examined before and after the switch to FO. The switch from a SO IVLE to FO IVLE in was associated with improvement in serum TG and VLDL concentrations, a significant increase in serum omega-3 fatty acids (*i.e.*, EPA, DHA), and a decrease in serum omega-6 fatty acids (*i.e.*, AA) [114].

Potential complications of FO IVLE have been reported. Mallah *et al.* described Burr cell anemia in an infant receiving FO monotherapy [129]. This resolved with discontinuation of the emulsion. Another possible explanation for the development of burr cell anemia is the association of preexisting liver disease and the presence of burr cells [130]. Another case report evaluating liver biopsy findings in 2 children treated with FO monotherapy failed to draw any meaningful conclusions due to the timing of the biopsies in relation to the start of treatment with FO [131]. Dimmitt *et al.*, in their analysis of 16 infants with IFALD treated with FO monotherapy, had more definitive findings; 6 patients had more than one biopsy and 3 patients had biopsies 6 months or longer after starting the fish oil monotherapy [132]. All biopsies showed evidence of fibrosis before initiating FO. In the patients with more long-term post-FO supplementation biopsies available, two showed resolving hepatic fibrosis and four showed no progression of fibrosis. This demonstrates the importance of timing of when the biopsies are obtained in the determination of disease progression and resolution.

### 1.7. Alternative IVLE Dosage Strategies

A second treatment strategy has emerged in which patients receive 50:50 doses by weight of both SO and FO for the treatment of IFALD [133]. The rationale for this approach was to prevent the theoretical risk of developing EFAD with FO monotherapy. In 12 patients treated with this combination therapy of 1 g/kg/day of each fat source (total 2 g/kg/day), no patients succumbed due to liver failure but 3 patients did ultimately go on to liver transplant. Moreover, 5 other patients required the discontinuation of SO and received FO as monotherapy as previously described in order for serum bilirubin levels to normalize. 

Another approach uses a combination IVLE that contains 30% SO, 30% MCT (as coconut oil), 25% olive oil, and 15% FO. The total amount of FO in this IVLE blend is significantly less than the FO monotherapy described above. In one retrospective cohort comparison over 6 months, IFALD-affected children were initiated on this combination therapy *vs.* remaining on SO IVLE. The median bilirubin at the outset was 8.36 mg/dL for the combination therapy group *vs.* 5.32 mg/dL for the SO IVLE group. At the end of 6 months, 5 of 8 children *vs.* 2 of 9 patients experienced resolution of jaundice with corresponding decreased of bilirubin of 5.79 mg/dL in the combination therapy group *vs.* an increase of bilirubin by 4.62 mg/dL in the SO group (*P* = 0.02) [134]. This suggests that the role of this lipid formulation may be less efficacious than a pure FO IVLE in the treatment of IFALD. Another study observed that the combination IVLE was well-tolerated with the only significant difference in outcomes being that the combination oil group had lower serum GGTP levels (107.8 ± 81.7 *vs.* 188.8 ± 176.7 IU/L, *P* < 0.05). The relative increase in body weight was not significant, however, there was an overall increase in serum omega-3:omega-6 ratio and α-tocopherol levels [135]. One double-blind RCT to assess the efficacy and safety of this IVLE in children receiving home parenteral nutrition (HPN) has been conducted. Subjects were randomized to the SO/MCT/olive oil/FO combination as described above or a standard SO IVLE [136]. The emulsions were infused 4 to 5 times per week at a goal dose of 2 g/kg/day. The changes in the total bilirubin levels between the initial and final values were significantly different between the two groups (1.5 ± 2.4 *vs.* 2.3 ± 3.5 µmol/L, *P < *0.01), with the children receiving the combination product having lower levels in comparison to the reference SO IVLE group. Similar findings in serum omega-3:omega-6 ratio and α-tocopherol status were also found in the combination IVLE group.

The reduction of SO IVLE has been suggested to contribute to the improvements in hepatic function, and the subsequent recommendation has been to reduce the dose to 1 g/kg/day [137]. Recent evidence, however, has since refuted this claim [138,139]. Rubinos *et al.* evaluated patients with IFALD at a tertiary care center who were either treated with a trial of SO IVLE prior to fish oil monotherapy or direct treatment with FO monotherapy [138]. This study found that the reduction of SO IVLE was insufficient in the treatment of IFALD. Nehra *et al*. studied whether the intravenous lipid emulsion dose: 1 g/kg/day and 2–3 g/kg/day affected the incidence of cholestasis based on the prior hypothesis [139]. There was no difference in time to cholestasis, therefore, SO IVLE reduction was inadequate in the prevention or delay of onset of cholestasis. Of note, once patients started therapy with FO, the time to resolution of cholestasis was faster in the low dose 1 g/kg/day SO lipid group with the mean time to normalization of direct being 41.4 ± 39.7 days compared with 52.6 ± 27.2 days for the 2 to 3 g/kg/day group (*P* = 0.95). Moreover, all the patients in this study who were transitioned to FO monotherapy subsequently developed biochemical resolution of their cholestasis. These findings suggest that in patients with pre-existing IFALD, FO monotherapy may be more efficacious than the SO/FO oil blend and that the combination of SO and FO may actually delay recovery from IFALD.

## 2. Conclusions

FO has shown dramatic improvement in patients with IFALD. While the complete mechanism of IFALD and its optimal treatment remain to be elucidated through further studies, FO IVLE has offered promising advances and improvement in this patient population.
